# Understanding the Link Between Hormonal Changes and Gingival Health in Women: A Review

**DOI:** 10.7759/cureus.85270

**Published:** 2025-06-03

**Authors:** Sidra Tul Muntaha Jawed, Khadeeja Tul Kubra Jawed

**Affiliations:** 1 Dental Department, Gulf Medical University, Dubai, ARE; 2 Surgical Department, Dubai Health Authority, Dubai, ARE

**Keywords:** estrogen, gingival inflammation, hormonal changes, periodontal health, progesterone

## Abstract

Sex hormones, particularly estrogen and progesterone, undergo continuous fluctuations throughout a woman's life, beginning at puberty and extending through the menstrual cycle, pregnancy, and menopause. These hormonal variations significantly influence gingival health, leading to various periodontal conditions. During puberty, elevated levels of estrogen and progesterone enhance blood circulation to the gingival tissues, increasing their sensitivity to plaque and resulting in puberty gingivitis. This condition is characterized by gingival enlargement, redness, and bleeding. Throughout the menstrual cycle, hormonal fluctuations can cause menstrual gingivitis, presenting as inflamed and bleeding gums, typically occurring prior to menstruation and subsiding thereafter. Pregnancy induces substantial hormonal changes, with heightened estrogen and progesterone levels exacerbating gingival inflammation. This often leads to pregnancy gingivitis, marked by swelling, bleeding, and tenderness of the gums. In some cases, a localized overgrowth known as a pyogenic granuloma or "pregnancy tumor" may develop on the gingiva. The use of oral contraceptives, which alter hormonal levels, has been associated with increased gingival inflammation and exudate, similar to the effects observed during pregnancy. Menopause brings a significant decline in estrogen levels, leading to various oral health issues such as dry mouth (xerostomia), a burning sensation in the mouth, and an increased risk of osteoporosis affecting the alveolar bone supporting the teeth. These changes contribute to a heightened susceptibility to periodontal diseases in post-menopausal women. The influence of estrogen and progesterone on the immune response, oral microbial composition, bone density, and enzymes like collagenase plays a crucial role in modulating gingival inflammation and the risk of periodontal diseases. Understanding these hormonal impacts is essential for developing effective prevention and treatment strategies for maintaining optimal gingival health in women across different life stages.

## Introduction and background

Men's and women's health have similarities, but women's health undergoes unique transformations that require specific attention, particularly regarding the effects of hormonal fluctuations on overall and oral health. Hormonal fluctuations primarily affect the gingiva and the periodontium within the oral cavity, often leading to changes in tissue response and susceptibility to inflammation [1].

The gingiva is defined as the mucosal part of the periodontium, which is attached to the tooth and serves to protect the tissues underneath from the influences of the oral environment [2]. Gingivitis is the inflammation of the gingiva and is manifested clinically as redness, swelling of the marginal gingiva, and bleeding on probing [2]. Gingival inflammation is a widespread disease. In 2010, its prevalence ranged between 50% and 94% among the adult population in the United States [3]. However, gingival inflammation often does not convert into a destructive periodontal disease [3].

Health has been defined by the American Academy of Periodontology (AAP) as "the condition of a patient when there is function without evidence of disease or abnormality." The health of the periodontium has been defined in different ways throughout the years, most commonly as the absence of disease. For instance, a healthy periodontium can be defined as the absence of progressive attachment loss around the tooth, in addition to the presence of proper occlusal functions [3]. The periodontal health is characterized by the absence of any clinical signs reflecting either past or present gingival and periodontal disease, and any tissue status outside the normal range [3]. However, this idealistic concept of a clinically perfect periodontium implies that even minimal deviations from a healthy status may be classified as a disease, making the term periodontal health relative [3].

The periodontium is a structure that includes the gingiva, bone, cementum, and periodontal ligament [4,5]. The periodontal disease prevalence ranges between 15%-17% and 5%-36% of the adult population in Hong Kong and the United States, respectively, which renders it a major health problem [6]. Periodontal disease can severely affect the quality of life of patients. About 22% of patients suffering from periodontitis report difficulties with chewing, diminished food flavor, and interruptions during meals [6]. Over 20% of patients report avoiding social outings because of their oral and periodontal issues [7]. The severity of the periodontal disease is assessed using several parameters, including bleeding on probing (BOP), probing pocket depth (PPD), clinical attachment level (CAL), halitosis, and tooth mobility [6].

Oral and periodontal health can be influenced by various factors, including systemic diseases, systemic conditions, immune deficiencies, stress and psychological conditions, and hormonal changes [3,6]. Sex hormones in women, such as estrogen and progesterone, fluctuate throughout their lives [8]. These hormones are also called steroid hormones, and they have several steroid receptors in the body [8]. The oral cavity and the periodontium are among the sites where these receptors are found. Therefore, fluctuations in the levels of these hormones, particularly during puberty, the menstrual cycle, pregnancy, and menopause, exert changes in the oral cavity and negatively impact its health. For instance, in pregnancy, pregnant women experience several changes, including physiological changes, hormonal changes, nutritional behavior changes, metabolic changes, and lifestyle changes [9]. As a result of these changes, gingival and mucosal alterations occur as well. The consequences of gingival and periodontal inflammation extend beyond the oral cavity; the inflammatory mediators cross the placental barrier and affect the developing embryo [9]. This emphasizes the importance of understanding how hormonal changes impact oral health in women throughout different stages of their lives. This review article aims to explore the connection between the hormonal fluctuations that women experience during different stages of their life and their gingival and periodontal health.

## Review

Methodology

This narrative review was based on a comprehensive literature search conducted from January 20 to January 24, 2025, across multiple databases: DynaMed, ScienceDirect, PubMed, Wiley Library, MDPI, Oxford Academic, BMC, and Cochrane. The search strategy used Medical Subject Headings (MeSH) and Boolean operators: (“Sex Hormones” OR “Estrogens” OR “Progesterone”) AND (“Gingival Diseases” OR “Periodontal Diseases”) AND (“Puberty” OR “Menstrual Cycle” OR “Pregnancy” OR “Menopause” OR “Oral Contraceptives”) AND (“Pregnancy Complications, Dental” OR “Pyogenic Granuloma” OR “Xerostomia” OR “Osteoporosis” OR “Alveolar Bone Loss”) AND (“Collagenase” OR “Immune System Phenomena” OR “Oral Microbiome” OR “Women’s Health”). These terms targeted studies examining the effect of hormonal changes on gingival and periodontal health in women. A manual search using Google Scholar and a review of reference lists from selected articles were also performed to identify additional relevant studies. No restrictions were applied regarding publication date, language, participant age, or type of publication.

Discussion 

Hormonal Changes Across Life Stages

**Figure 1:**
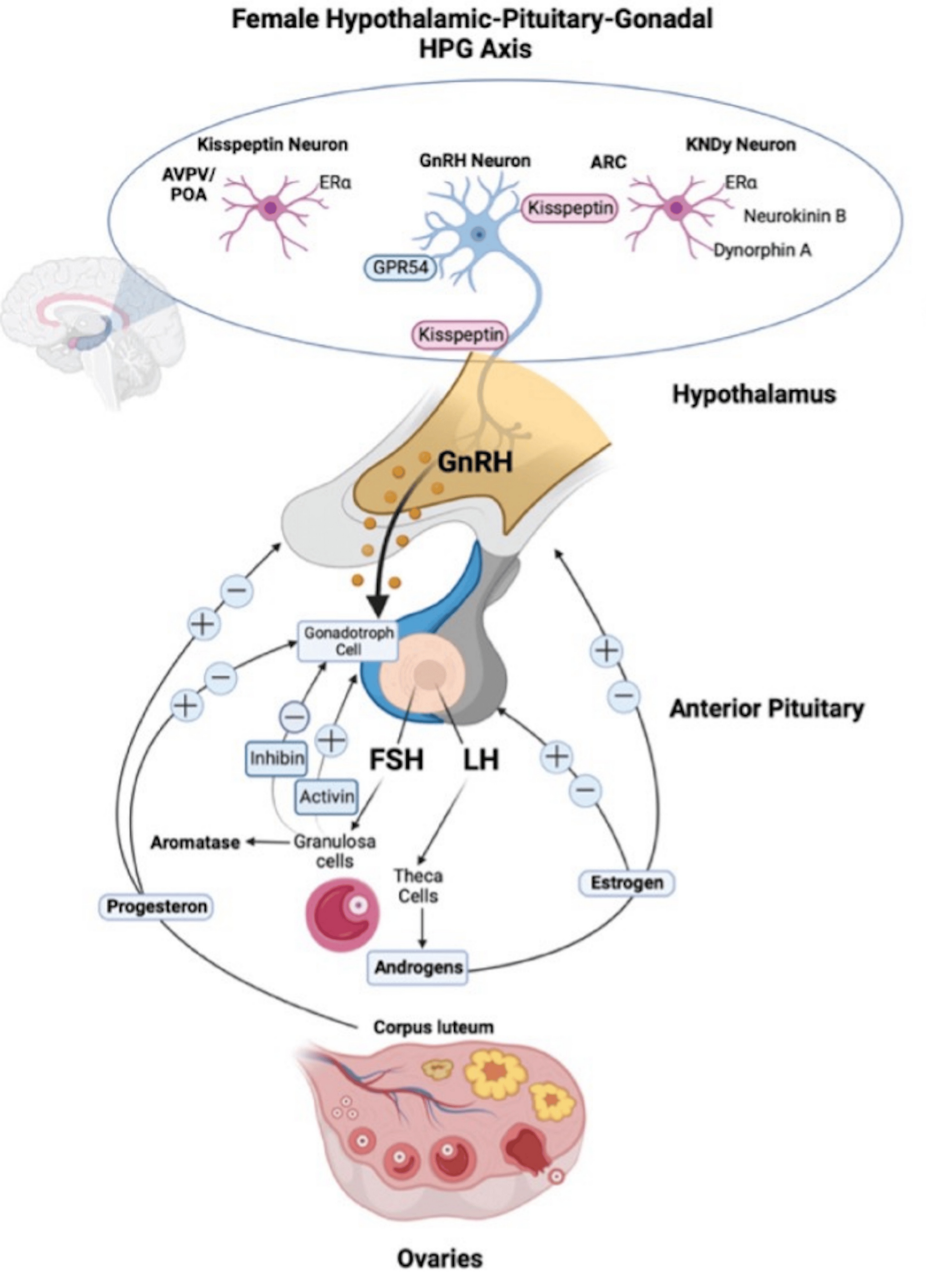
Female hypothalamic-pituitary-gonadal axis. AVPV/POA: anteroventral periventricular nucleus/preoptic area, ERa: estrogen receptor alpha, GnRH: gonadotropin-releasing hormone, GnRH neuron: gonadotropin-releasing hormone neuron, ARC: arcuate nucleus, KNDy neuron: kisspeptin/neurokinin B/dynorphin neuron, FSH: follicle-stimulating hormone, LH: luteinizing hormone, GPR54: kisspeptin receptor (binds kisspeptin to activate GnRH release), HPG: hypothalamic-pituitary-gonadal. Reproduced with permission from Tammasse et al. [10]. This open-access article is distributed under the terms of the Creative Commons Attribution (CC BY 4.0) license.

To understand hormonal changes, we must comprehend how the hormones are secreted and how they fluctuate. The cycle starts by releasing the follicle-stimulating hormone (FSH) from the hypothalamus. The FSH recruits ovarian follicles; these follicles lead to the rise of estrogen levels. Estrogen exerts negative feedback on the hypothalamic-pituitary-gonadal axis, which decreases the FSH levels. One of the recruited follicles becomes the dominant follicle as a result of estrogen levels peaking and progesterone levels exhibiting a small rise. Then, the follicle ruptures and ovulation occurs, leading to a significant increase in progesterone levels. If fertilization does not occur, then estrogen and progesterone levels drop [8]. These constant fluctuations in hormones start from puberty and extend to menopause (Figure 1).

Puberty: Puberty is the period when children mature and become adults. It is associated with physical, physiological, and hormonal changes [11]. The significant increase in the secretion of sex hormones, such as estrogen and progesterone, affects the periodontium. The gingival tissues respond to these increases by changing their microenvironment; hence, the microorganisms that used to capitalize on that environment also change [11]. One of the significant clinical manifestations of puberty, which highlights the impact of sex hormones on gingival tissues, is puberty gingivitis [11]. It peaks from the age of nine to the age of 14 [12]. The characteristics of puberty gingivitis include a significant increase in gingival inflammation without an increase in accumulated plaque [13,14]. Higher populations of gram-negative rods in the subgingival microflora with certain species of bacteria, such as *P. intermedia* and *Capnocytophaga* species [1,15], an increase in blood circulation in the terminal capillaries, leading to increased bleeding tendencies [16], increased vascular permeability, and endothelial damage [12]. However, sex hormone levels remain a secondary reason for gingivitis. Estrogen and progesterone exacerbate the already existing situation [17].

Menstrual cycle: The menstrual cycle is defined as the beginning of the first day of menses and continuing till the day before the next menses; this process occurs on average every 28 days [1,18]. It is characterized by the onset of increased production of estrogen and progesterone [1,11]. The menstrual cycle consists of two phases. The first phase is the follicular phase, where the estrogen levels peak two days before ovulation; after ovulation, the progesterone levels peak and then drop before menstruation [1]. These fluctuations throughout the menstrual cycle caused numerous significant alterations in the periodontium, such as gingivitis intermenstrualis, which is a condition in which the gingiva becomes red, swollen, and hemorrhagic before menstruation [11].

Progesterone increases the permeability of the microvasculature, which alters collagen production in the gingiva, increases folate metabolism, and stimulates the production of inflammatory mediators such as polymorphonuclear leukocytes [1]. Hence aggravates gingival inflammation, bleeding, swelling, and gingival exudates, and causes minor tooth mobility [1]. Moreover, when progesterone peaks, recurrent aphthous ulcers, herpes labialis lesions, and candida infections may prevail in the oral (NA1) cavity [1]. As a result, most gingival changes happen during the ovulation period, for example, the crevicular fluid increases by 20% in 75% of women during the ovulation period [19].

Pregnancy: During pregnancy, estrogen and progesterone are the dominant hormones in the body. Their levels start increasing from the second month till the eighth month and peak in the first and second trimesters [11]. This often leads to alterations in the periodontium. These alterations are commonly present as pregnancy gingivitis and pyogenic granuloma. Approximately 35% to 100% of women experience pregnancy gingivitis [11].

The characteristics of pregnancy gingivitis include increased infection susceptibility, depressed antibody production, decreased neutrophil chemotaxis, and increased prostaglandin production [16]. The clinical features of pregnancy gingivitis are increased probing depth, gingival edema [11], increased BOP, erythema, and tooth mobility [1,19-21]. The anterior region of the oral cavity is affected the most by pregnancy gingivitis [1]. The severity of the inflammation begins in the second month of pregnancy and increases gradually through the remaining eight months; then it significantly decreases after labor as the sex hormone levels decrease [1].

**Figure 2:**
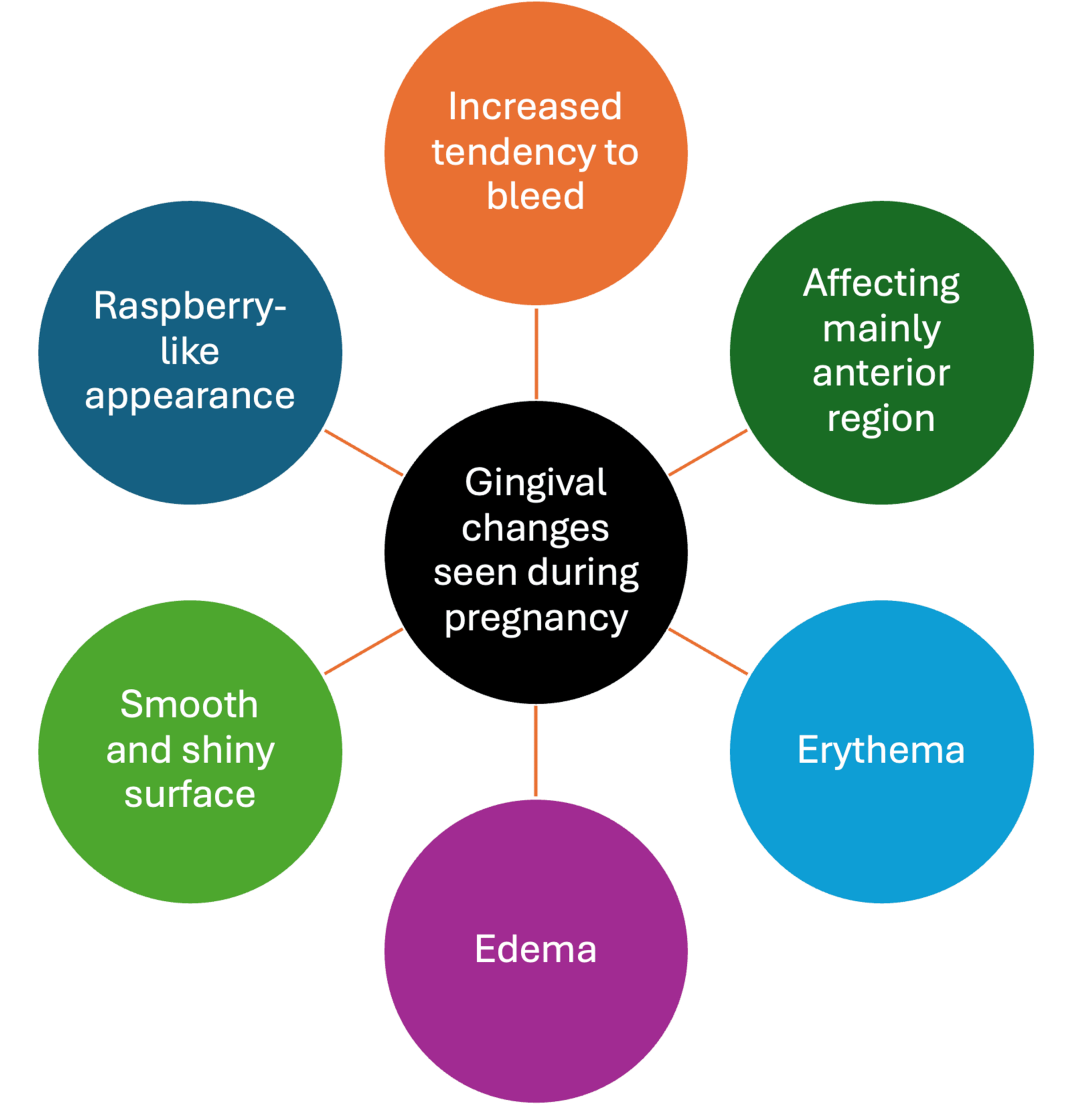
Gingival changes seen during pregnancy. Image credits to author: Sidra Tul Muntaha Jawed.

Pyogenic granuloma or pregnancy tumor is an inflammatory gingival lesion that appears in the first or second trimester due to a combination of factors such as plaque and altered hormonal balance [11]. About 0.2% to 9.6% of pregnant women may experience pyogenic granuloma [1,22]. Its most common site is the gingiva, with 70% of the cases, followed by the tongue, lips, buccal mucosa, and palate [1,22]. Pyogenic granulomas often affect the anterior part of the gingiva [23,24]. The tumor bleeds easily, is painful, and interferes with mastication [11]. The lesion results from the increased levels of estrogen and progesterone and their influence on the immune system. Moreover, progesterone inhibits the collagenase enzyme in the gingiva, hence affecting the gingival vasculature [11, 25] (Figure 2).

Menopause: The World Health Organization (WHO) defines menopause as the permanent termination of menstruation for 12 months due to the cessation of ovarian follicular activity [26,27]. Menopause is a physiologic phenomenon but can be induced artificially by radiation, chemotherapy, and surgery [26]. It usually begins in the fourth decade, often between the ages of 45 and 55 years old [1,11,26]. The significant decline in estrogen and follicle functions causes various physical and psychological changes; among these changes are the oral manifestations [11].

During the premenopausal phase, women may encounter a range of oral manifestations associated with hormonal fluctuations, particularly the fluctuations involving estrogen and progesterone. These hormonal variations can significantly affect vascular permeability and the immune response within the oral mucosa, rendering premenopausal women more vulnerable to gingival inflammation, increased bleeding upon probing, and pronounced reactions to local irritants. While tooth loss is more pronounced in post-menopausal women, the early signs of periodontal disease may already be evident in premenopausal women, particularly in the context of inadequate oral hygiene practices. Furthermore, hormonal changes during this period may contribute to symptoms such as xerostomia and altered taste perception, which can adversely impact both the quality of life and emotional well-being of affected individuals [28].

Oral manifestations in menopause are represented in four main symptoms, which are menopausal gingivostomatitis, osteoporosis, burning mouth syndrome, and xerostomia. Menopausal gingivostomatitis is characterized by dry, abnormal paleness or redness, and shiny gingiva and oral mucosa with an increased bleeding tendency [1,11].

Osteoporosis is a condition in which the mineral density in bones and bone mass decrease, accompanied by deformity, pathological fractures, exaggerated bone resorption, and reduced bone formation [29,30]. The WHO has categorized osteoporosis into two categories: primary and secondary. Furthermore, primary osteoporosis is subdivided into three types: type I is post-menopausal osteoporosis, type II is age-related osteoporosis, and type III is idiopathic osteoporosis [29,30]. In post-menopausal osteoporosis, the mandible is more susceptible than the maxilla [11]. This condition is attributed to the decline of estrogen levels after cessation of menstruation and follicular activity. This led to a decrease in osteoblastic physiological activities compared to steady levels of osteoclastic activity [29,31]. Osteoporosis is accompanied by crestal bone loss, which accelerates the existing periodontal diseases and hinders wound healing, which leads to tooth mobility [1,16].

**Figure 3:**
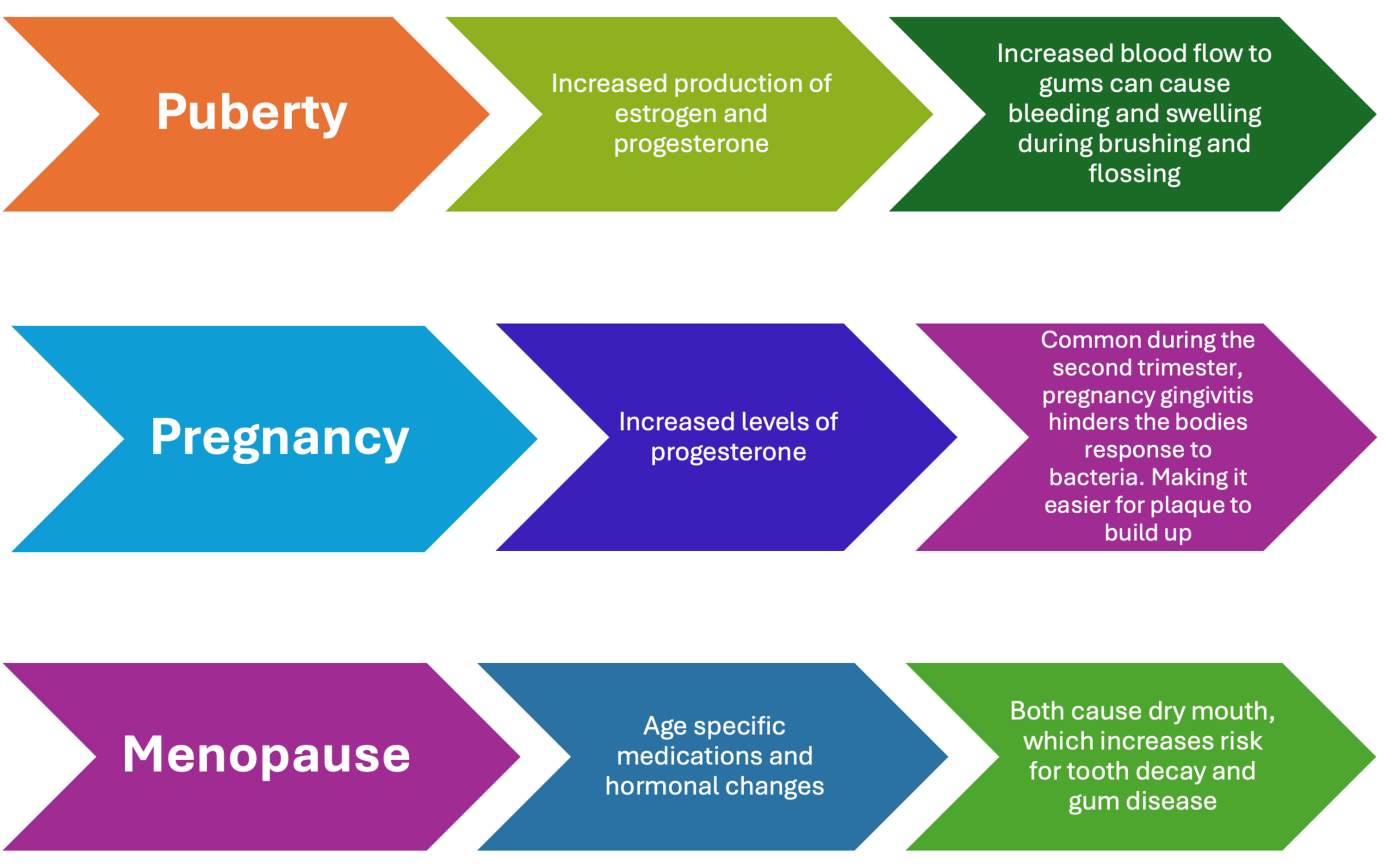
Woman’s oral health timeline. Image credits to author: Sidra Tul Muntaha Jawed.

One of the oral manifestations of menopause is burning mouth syndrome. Burning mouth syndrome is a condition in which the patient experiences burning pain with no evidence of medical or dental causes [26]. It affects the tongue, lips, and gums and is often bilateral. Patients with burning mouth syndrome suffer from taste alterations, bad breath, difficulty swallowing, and facial pain [26]. Meurman et al. studied the link between menopause and oral discomfort in 149 women. Forty-three percent of perimenopausal women experienced oral discomfort, while only 6% of premenopausal women suffered from it [32]. Another oral manifestation associated with estrogen reduction is xerostomia, which results from the reduction of salivary gland secretion [1,26,33] (Figure 3).

Oral Contraceptives and Hormonal Therapy

Oral contraceptives are drugs that depend on gestational hormones (estrogen and progesterone) to stimulate the state of pregnancy and thus prevent ovulation [1,18]. Gingival tissues respond exaggeratedly to progesterone and estrogen. The inflammation ranges from mild edema and erythema to hemorrhage and hyperplasia [1]. Oral contraceptives increase gingival fluid by 50%, increase Bacteroides species by 16-fold, and increase localized osteitis by two-fold following extractions because oral contraceptives affect clotting factors and cause clot lysis [1]. Additionally, Candida species are higher in women receiving oral contraceptives [34]. A study conducted by Güncü et al. [1] has shown that oral contraceptives cause spotty melanotic pigmentation and some irregular brownish marks, especially in areas exposed to sun radiation [11,35]. Furthermore, long-term use of oral contraceptives decreases the risk of osteoporosis [11]. However, current oral contraceptives have low doses of steroidal hormones and, therefore, have fewer oral side effects [19]. Older high-dose oral contraceptives (≥50 µg ethinylestradiol) were frequently associated with gingival inflammation, increased plaque accumulation, and periodontal degradation. These effects were attributed to hormone-induced changes in vascular permeability and immune response. Modern combined oral contraceptives contain lower doses of ethinylestradiol, typically between 10 and 35 µg. This reduction has led to fewer oral side effects. However, mild gingival sensitivity or inflammation may still occur, particularly in individuals with poor oral hygiene or existing periodontal conditions. While better tolerated, low-dose formulations do not completely eliminate the risk of oral complications [36,37].

Mechanisms Linking Hormonal Changes to Gingival Health

Hormonal fluctuations have an impact on immune modulation, inflammatory response, subgingival microflora [11], and vascular permeability [2]. For instance, estrogen decreases keratinization, decreases the efficiency of the epithelial barrier, reduces fibroblast proliferation, protein production, and T-cell-mediated inflammation, inhibits polymorphonuclear leukocyte chemotaxis, and suppresses interleukin-6 (IL-6) [11]. Whereas progesterone increases vascular permeability, reduces glycosaminoglycan synthesis, alters collagen production, reduces IL-6 production as well, and increases folate metabolism, which negatively affects the repair rate of the periodontal tissues and promotes prostaglandin E2 synthesis [11]. Sex hormones have receptors in the periodontal tissues, which render them influential in the inflammatory state of these tissues, as they consider the periodontium a target tissue [11].

For example, during the menstrual cycle, tumor necrosis factor-alpha (TNF-α) fluctuates and peaks before ovulation, while both interleukin-2 (IL-2) and IL-6 show no fluctuations before menstruation. However, IL-6 has a negative correlation with estrogen and progesterone [38]. These cytokines are crucial in the initiation, progression, and modulation of host immunity during periodontal diseases [38]. TNF-α stimulates bone resorption and collagenase enzyme production and hinders periodontium tissue repair [38]. The presence of these cytokines in the subgingival tissues indicates the presence of subclinical inflammation. These cytokines are very sensitive to hormonal changes; therefore, whenever the oral hygiene level drops, inflammation is exaggerated. As the presence of low plaque and good oral hygiene limits the influence of sex hormones on the gingiva [38].

Furthermore, during pregnancy, women’s eating habits change, their food intake increases, and their oral hygiene and dental visits decrease [23,39]. Poor oral hygiene during pregnancy is attributed to pregnancy symptoms such as frequent nausea and vomiting. This poor oral hygiene, alongside the increased levels of estrogen and progesterone, causes inflammation, gingivitis, periodontitis, and, in a few cases, pyogenic granuloma. A study conducted by Gaetti-Jardim et al. [40] shows that only 5.88% of pregnant women sought professional dental follow-up, only 9% sought professional preventive treatments, and 60% of pregnant women did not seek any type of professional treatment or follow-up [23].

Clinical Implications and Preventive Strategies

The development of gingivitis and periodontal diseases results from the accumulation of plaque in the mouth for several weeks without removal; this leads to disruption of the symbiosis between the biofilm in the oral cavity and the host’s immune system. As a consequence, dysbiosis occurs, leading to an imbalance in microbial populations that subsequently target the periodontium, resulting in inflammation. Various systemic factors can influence this microbial symbiosis, including hormonal fluctuations, the administration of immunosuppressive medications, and underlying systemic health conditions [19].

Understanding the influence of hormonal fluctuations on periodontal health has critical clinical implications for dental professionals and healthcare providers. Because estrogen and progesterone impact gingival inflammation, vascular permeability, and microbial composition, specific life stages--including puberty, pregnancy, menstruation, and menopause--pose unique challenges in maintaining optimal oral health. The development of tailored preventive strategies and treatment approaches is essential to mitigate the risk of periodontal disease and improve overall health outcomes for women [41,42].

Importance of preventive care: Preventive dental care plays a crucial role in managing hormonally influenced gingival and periodontal changes. Regular dental visits, thorough oral hygiene practices, and professional periodontal maintenance are essential at all stages of a woman's life. The following preventive measures should be emphasized for different life stages:

Puberty: Education about proper brushing and flossing techniques should be reinforced in adolescent patients, as hormonal fluctuations increase susceptibility to plaque-induced gingival inflammation. Antimicrobial mouthwashes and professional cleanings may help manage puberty gingivitis [31-34,41-43].

Menstrual cycle: Women experiencing menstrual gingivitis should be advised to maintain rigorous oral hygiene, including daily flossing and the use of chlorhexidine mouthwash. Dentists should also educate patients about temporary gingival swelling and increased bleeding tendencies during the luteal phase [43].

Pregnancy: Pregnant women should be encouraged to schedule additional dental visits for professional cleanings, particularly during the second trimester, when pregnancy gingivitis often peaks. Scaling and root planning should be performed as needed to reduce the bacterial load and inflammation. Patient education should highlight the relationship between periodontal health and adverse pregnancy outcomes such as preterm birth and low birth weight [44].

Menopause: Post-menopausal women should be screened for osteoporosis-related alveolar bone loss, and preventive strategies, such as calcium and vitamin D supplementation, should be recommended. Patients should also be advised to stay hydrated and use saliva substitutes if they experience xerostomia [45].

Management Strategies for Hormonal Gingival Changes

Beyond preventive care, specific management strategies can be employed to address the unique challenges posed by hormonal fluctuations in different stages of life (Table 1).

**Table 1:** Dental hygiene strategies for women at different life stages.

Phase of therapy	Adolescence	Womanhood	Menopause/post-menopause
Initial (non-surgical) therapy	Oral hygiene education (gingivitis prevention), D4346: scaling in the presence of gingivitis, daily antimicrobial mouth rinse	Oral hygiene education (pregnancy gingivitis), early periodontal examination for pregnancy, intraoral exam: pyogenic granulomas	Oral hygiene education (menopausal gingivostomatitis), recommendations for xerostomia/burning mouth (xylitol rinses, lozenges, sprays)
Surgical/non-surgical therapy	—	Surgical and non-surgical periodontal therapies	Surgical and non-surgical periodontal therapies (if indicated)
Risk factor identification and medical history	Medical history: oral contraceptive use (type/dosage), schedule dental visits before menstruation	Interprofessional collaboration and referrals, medical history: pregnancy status	Medical history review and interprofessional collaboration, consider hormone replacement therapy (benefits and limitations)
Maintenance therapy	Educate on the importance of regular dental visits	Three-month recall post-pregnancy	Regular dental visits (may require three- to four-month periodontal recalls), nutritional counseling

Periodontal Therapy and Non-surgical Interventions

Scaling and root planning: Non-surgical periodontal therapy is the first-line treatment for pregnancy gingivitis, puberty gingivitis, and menopausal gingival inflammation. Regular cleanings and subgingival debridement can significantly reduce bacterial load and inflammation [46].

Antimicrobial therapy: 0.1% Chlorhexidine mouthwash and localized antibiotic therapy, such as doxycycline gel, can help manage persistent gingival inflammation, particularly in patients with pregnancy gingivitis or oral contraceptive-induced gingivitis [47,48].

Saliva substitutes and hydration therapy: In menopausal women experiencing xerostomia, artificial saliva products and increased fluid intake can help alleviate discomfort and reduce the risk of caries and periodontal disease [49].

Patient Education and Behavioral Modifications

Oral hygiene instruction: Patients should receive individualized education on proper brushing techniques, including the use of soft-bristled toothbrushes and interdental cleaning devices. Electric toothbrushes may be beneficial for individuals with dexterity issues, such as post-menopausal women [50].

Nutritional counseling: A diet rich in vitamins C and D, calcium, and omega-3 fatty acids supports periodontal health. Pregnant and post-menopausal women should be advised on nutrient intake to maintain bone density and reduce inflammation [51].

Smoking cessation programs: Smoking is a major risk factor for periodontal disease and exacerbates the effects of hormonal changes on gingival health by impairing immune function, reducing blood flow to the gums, and hindering healing processes. Smokers have more bone loss, attachment loss, and tooth loss compared to non-smokers. Patients should be encouraged to quit smoking, especially during pregnancy and menopause, to reduce inflammation and improve treatment outcomes [52].

Hormone-Related Therapeutic Interventions

Hormone replacement therapy (HRT): Man et al. [53] suggested in their study that HRT may help mitigate osteoporosis-related alveolar bone loss and reduce gingival inflammation in post-menopausal women. However, HRT's impact on periodontal disease remains controversial, and further research is needed to determine its long-term efficacy and safety [53].

Alternative hormonal therapies: Phytoestrogens (plant-derived estrogen-like compounds) found in soy products have been proposed as potential alternatives to HRT for managing menopausal symptoms. Some research suggests that phytoestrogens may have a protective effect on alveolar bone loss and periodontal health, though additional clinical trials are needed [54].

Interdisciplinary Collaboration in Women's Health and Periodontal Care

Given the systemic implications of periodontal disease and its link to conditions such as cardiovascular disease, diabetes, and adverse pregnancy outcomes, an interdisciplinary approach is essential. Healthcare providers, including gynecologists, endocrinologists, obstetricians, and periodontists, should work together to optimize patient care.

Obstetric collaboration: Obstetricians should include oral health screenings in prenatal care and refer pregnant patients for periodontal evaluation if inflammation is present [55].

Endocrine considerations: Endocrinologists treating patients with polycystic ovary syndrome (PCOS) or osteoporosis should assess oral health risks and collaborate with dental professionals to mitigate periodontal complications [56].

Bone health assessments: Post-menopausal women undergoing osteoporosis treatment (e.g., bisphosphonates) should receive a dental evaluation before starting therapy, as bisphosphonates can increase the risk of medication-related osteonecrosis of the jaw (MRONJ) [57].

Special Considerations for Oral Contraceptive Users

Women using oral contraceptives may experience an increased risk of gingival inflammation due to elevated progesterone levels. While modern low-dose contraceptives have reduced these effects compared to earlier formulations, patients should still be advised on the importance of maintaining optimal oral hygiene. Additionally, oral contraceptives can alter clotting mechanisms, potentially increasing the risk of post-extraction complications such as localized osteitis (dry socket). Dentists should assess contraceptive use when planning surgical procedures and consider prophylactic measures such as the use of chlorhexidine mouthwash post-operatively [58].

Future Research and Emerging Therapies

While existing treatment strategies focus primarily on managing inflammation and reducing bacterial burden, emerging therapies may offer novel approaches to hormone-related periodontal disease. Future research should explore:

Targeted anti-inflammatory agents: Medications that modulate inflammatory mediators involved in hormonal gingival changes (e.g., TNF-α inhibitors) may provide more precise treatment options [59].

Regenerative periodontal therapy: Growth factors and stem cell therapy could potentially counteract alveolar bone loss associated with menopause [60].

Personalized medicine approaches: Genetic and hormonal profiling may allow for individualized periodontal treatment plans based on a patient's specific hormonal status and inflammatory response [61].

## Conclusions

Women experience hormonal fluctuations throughout their lives, beginning with puberty and continuing until menopause. These changes can significantly impact their overall health and well-being. Gingival and periodontal health are influenced by these hormonal variations at different stages of a woman's life. Specifically, progesterone and estrogen have been shown to alter the immune response and the oral biofilm, leading to exaggerated gingival inflammation. Understanding these hormonal impacts is essential for developing effective prevention and treatment strategies for maintaining optimal gingival health in women across different life stages.
